# A structured program for teaching pancreatojejunostomy to surgical residents and fellows outside the operating room: a pilot study

**DOI:** 10.1186/s12893-021-01101-w

**Published:** 2021-02-25

**Authors:** Kenichi Oshiro, Kazuhiro Endo, Kazue Morishima, Yuji Kaneda, Masaru Koizumi, Hideki Sasanuma, Yasunaru Sakuma, Alan Kawarai Lefor, Naohiro Sata

**Affiliations:** Department of Surgery, Jichi Medical University, 3311-1 Yakushiji, Shimotsuke, Tochigi 329-0498 Japan

**Keywords:** Pancreatojejunostomy, Surgical education, Simulation training, 3D model, Training program, Standardization

## Abstract

**Background:**

Pancreatojejunostomy (PJ) is one of the most difficult and challenging abdominal surgical procedures. There are no appropriate training systems available outside the operating room (OR). We developed a structured program for teaching PJ outside the OR. We describe its development and results of a pilot study.

**Methods:**

We have created this structured program to help surgical residents and fellows acquire both didactic knowledge and technical skills to perform PJ. A manual was created to provide general knowledge about PJ and the specific PJ procedure used in our institution. Based on questionnaires completed by trainers and trainees, the procedure for PJ was divided into twelve steps and described in detail. After creating the manual, we developed organ models, needles and a frame box for simulation training. Three residents (PGY3-5) and three fellows (PGY6 or above) participated in a pilot study. Objective and subjective evaluations were performed.

**Results:**

Trainees learn about PJ by reading the procedure manual, acquiring both general and specific knowledge. We conducted simulation training outside the OR using the training materials created for this system. They simulate the procedure with surgical instruments as both primary and assistant surgeon. In this pilot study, as objective assessments, the fellow-group took less time to complete one anastomosis (36 min vs 48 min) and had higher scores in the objective structured assessment of technical skill (average score: 4.1 vs 2.0) compared to the resident-group. As a subjective assessment, the confidence to perform a PJ anastomosis increased after simulation training (from 1.6 to 2.6). Participants considered that this structured teaching program is useful.

**Conclusion:**

We developed a structured program for teaching PJ. By implementing this program, learning opportunities for surgical residents and fellows can be increased as a complement to training in the OR.

## Background

A surgical procedure consists of multiple tasks. To complete the tasks effectively and safely, a surgeon must have both appropriate didactic knowledge and technical skills. Therefore, acquiring knowledge regarding procedures and training in technical skills are both essential. Challenging surgical procedures require surgeons to perform challenging tasks. Pancreatojejunostomy (PJ) is considered one of the most difficult and challenging surgical procedures in abdominal surgery. Difficulties with this procedure can lead to serious complications and poor outcomes [1, 2]. Pancreatic surgeons must acquire both advanced knowledge and refined technical skills to perform PJ.

For surgical education, the education system and specific programs are important. The education system for surgeons in Japan includes a 3-year surgical residency program which follows a 2-year internship (required of all medical school graduates in Japan). Trainees can then seek further training as fellows in a subspecialty. Usually at the PGY6 level or later, surgeons begin training in a particular subspecialty, similar to a fellowship in many countries. Surgical trainees usually work at teaching hospitals. Therefore, they perform surgery under the supervision of instructors and rarely perform surgery independently. Education for specific procedures is mainly done in the operating room (OR) in an apprenticeship manner. There are 24 residents in our surgical residency program, and there are nine fellows in gastrointestinal surgery in our hospital.

However, there are several problems associated with teaching the PJ procedure. First, there is no single standardized technique for this procedure. There are many variants of the PJ. There are differences in the way the procedure is performed among surgeons, even in the same institution [3]. Second, training opportunities in the OR are very limited. The number of cases of hepatobiliary and pancreatic surgery at each facility are relatively small. As the result, there are limited opportunities for trainees to operate as the primary surgeon, especially for a complex procedure such as PJ.

Patient safety must be considered when teaching this procedure. An effective training program is needed for the PJ procedure. To date, there are no appropriate programs with appropriate teaching materials for teaching the PJ procedure.

The goal of this study is to establish a teaching program for the PJ anastomosis outside the OR which complements training in the OR. In this paper, we first describe the development of a structured program with a procedure manual and physical simulation models as teaching materials to create a complete system for teaching PJ. Second, we report the results of a pilot study.

## Methods

In this study, a structured teaching program was developed first, and then a pilot study was conducted. In the development of the program and the pilot study, surveys were conducted anonymously. This pilot study was conducted to assess the structured program including the manual and the models. This study did not meet the definition of human research that requires research ethical review in our institution and the requirement for institutional review board approval was waived.

### Development of the structured teaching program

The development of this program is based on the framework of clinical assessment by Miller [4]. Miller described that learning could be ranked into the following four stages: knows (knowledge), knows how (competence), show how (performance) and does (action). It is well-known as the assessment pyramid of Miller. Our program is aiming to acquire “knows” and “knows how”.

The target trainees are surgical residents and fellows who have never performed a PJ anastomosis with the modified Blumgart technique. Many of these trainees are seeking advanced training in hepatobiliary pancreatic surgery.

The structured program consists of self-learning using a procedure manual and simulation training with inanimate models. For teaching materials, we first created a procedure manual. Following the manual, organ models, needles and a frame-box were developed for the program.

#### Creation of the procedure manual and standardization of the PJ technique

A procedure manual includes both general and specific knowledge regarding the PJ procedure. General knowledge in the manual includes preoperative risk assessment, a review of techniques used to perform a PJ and a discussion of postoperative complications. As specific knowledge, the manual specifically describes the modified Blumgart technique, which is the standard technique adopted at our institution for PJ in patients with soft pancreatic parenchyma and a non-dilated main pancreatic duct.

**Fig. 1:**
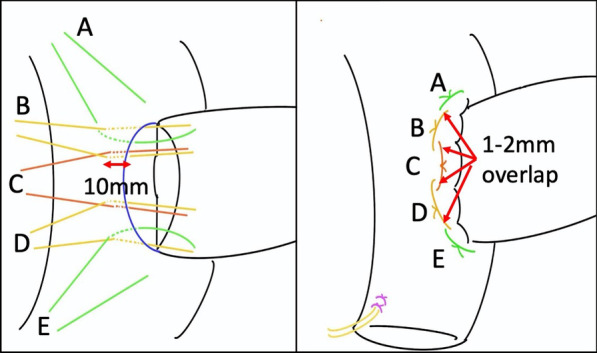
Schema for the final steps of the procedure. Every instruction is provided in words, numbers and illustrations. The purpose of these steps is to wrap the stump of the pancreatic parenchyma with the jejunum circumferentially. Technical tips include: Be careful to leave a ligation point on the jejunum. The assistant must help to maintain the wrapped shape with the jejunum. Be careful not to make the ligation too tight

We have a number of senior surgeons who serve as instructors, but we want to teach a single way to perform this procedure to our trainees. The wide range of variations in technique used make it difficult for instructors to teach one method for the procedure as baseline-level knowledge. To minimize technical differences among the instructors, the principals of a Delphi method were employed. We utilized a repeated questionnaire. First, a trainee, who had never performed the PJ, made a list of questions to instructors about PJ after going through materials including surgery videos, operative reports and references. The questions were collated and then given to a panel of six board-certified surgeons, all accredited by the Japanese society of hepato-biliary- pancreatic surgery. Responses were kept anonymous. The results of the first survey were compiled. Points of agreement and disagreement among instructors were identified. An additional questionnaire based on the results of first survey was then created and a second survey carried out. We repeated these anonymous surveys to obtain a basic consensus among our experts regarding technical differences. This allowed development of a procedure manual with a unified approach. In the procedure manual, we divided the PJ procedure into twelve steps and state the purposes of each step clearly (Table 1). The standardized procedure is described in as much detail as possible. Simple illustrations are also used to increase the level of understanding of the procedure (Fig. 1).

**Table 1:** Structured program for teaching pancreatojejunostomy outside the operating room

General knowledge of pancreatojejunostomy
Preoperative assessment of risk for development of a postoperative pancreatic fistula
Various techniques for pancreatojejunostomy
Postoperative complications
Management of postoperative pancreatic fistula
Steps in the pancreatojejunostomy technique (modified Blumgart method)
Preparation of the pancreas and the jejunum Preparation of the pancreas and the jejunum
Marking the jejunal serosa
Incision of the jejunal serosa
Incision and puncture of jejunal wall at the site of duct-to-mucosa anastomosis
Placement of an external stent
U-shaped sutures on the posterior side of the jejunum and pancreatic parenchyma (three central sutures)
U-shaped sutures on the jejunum and the pancreatic parenchyma (upper and lower edge sutures)
Duct-to-mucosa anastomosis
Suturing the anterior side of the jejunum (three central sutures)
Suturing the anterior side of the jejunum (Upper and bottom edge)
Ligation of U-shape sutures
Placement of a drain

#### Creation of teaching materials for simulating the surgical procedure

**Fig. 2:**
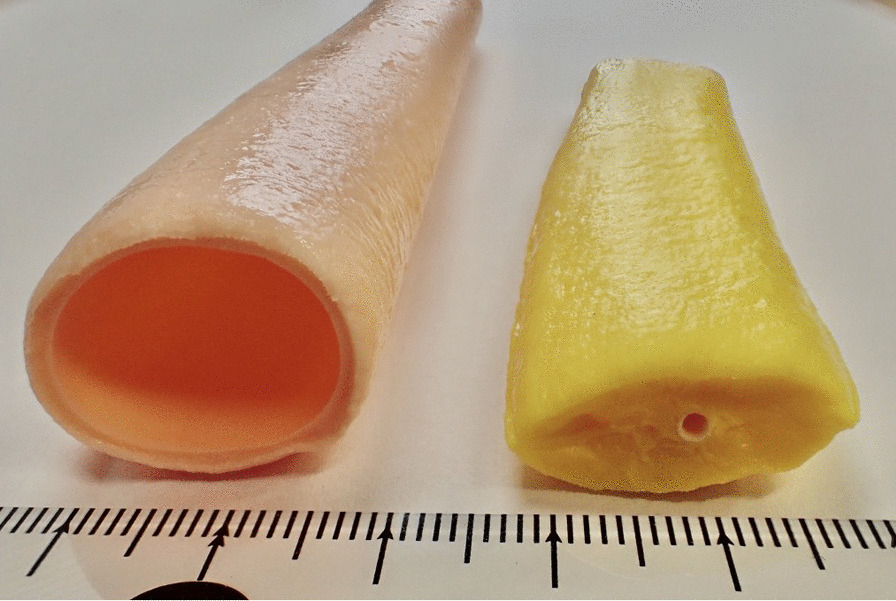
The organ models which used our simulation training. (Left) small bowel model. (Right) pancreatic model with non-dilated main pancreatic duct

Based on the manual that we created for the standard PJ technique, we developed teaching materials necessary for physical simulation training. We developed inanimate organ models including a pancreas model and a small bowel model. They were developed to meet the following two requirements. First, the models replicate the anatomical features required to perform simulation training. Second, the models must be usable for simulation training with regular surgical instruments, sutures and needles. The pancreas model replicates a pancreas with soft parenchyma and a non-dilated main pancreatic duct. The main pancreatic duct has a diameter of 1.5 mm and is located slightly dorsal and cranial to the center. The small bowel model has a diameter of approximately 2 cm and is 40 cm long. The models were made from polyvinyl chloride which has elasticity. The models were especially designed to contain fibers to reduce the incidence of splitting after suturing. One model set can be utilized for four anastomosis training sessions. We developed them in conjunction with FASOTEC Medical Engineering Company (Chiba, Japan) [5] (Fig. 2).

To reduce the costs of this training, we also developed needles for simulation training in conjunction with CROWNJUN Inc. (Tokyo, Japan). The needles are the same length, thickness, and curvature as the standard items utilized in our institution. Nylon was selected as the suture material to further reduce costs.

**Fig. 3:**
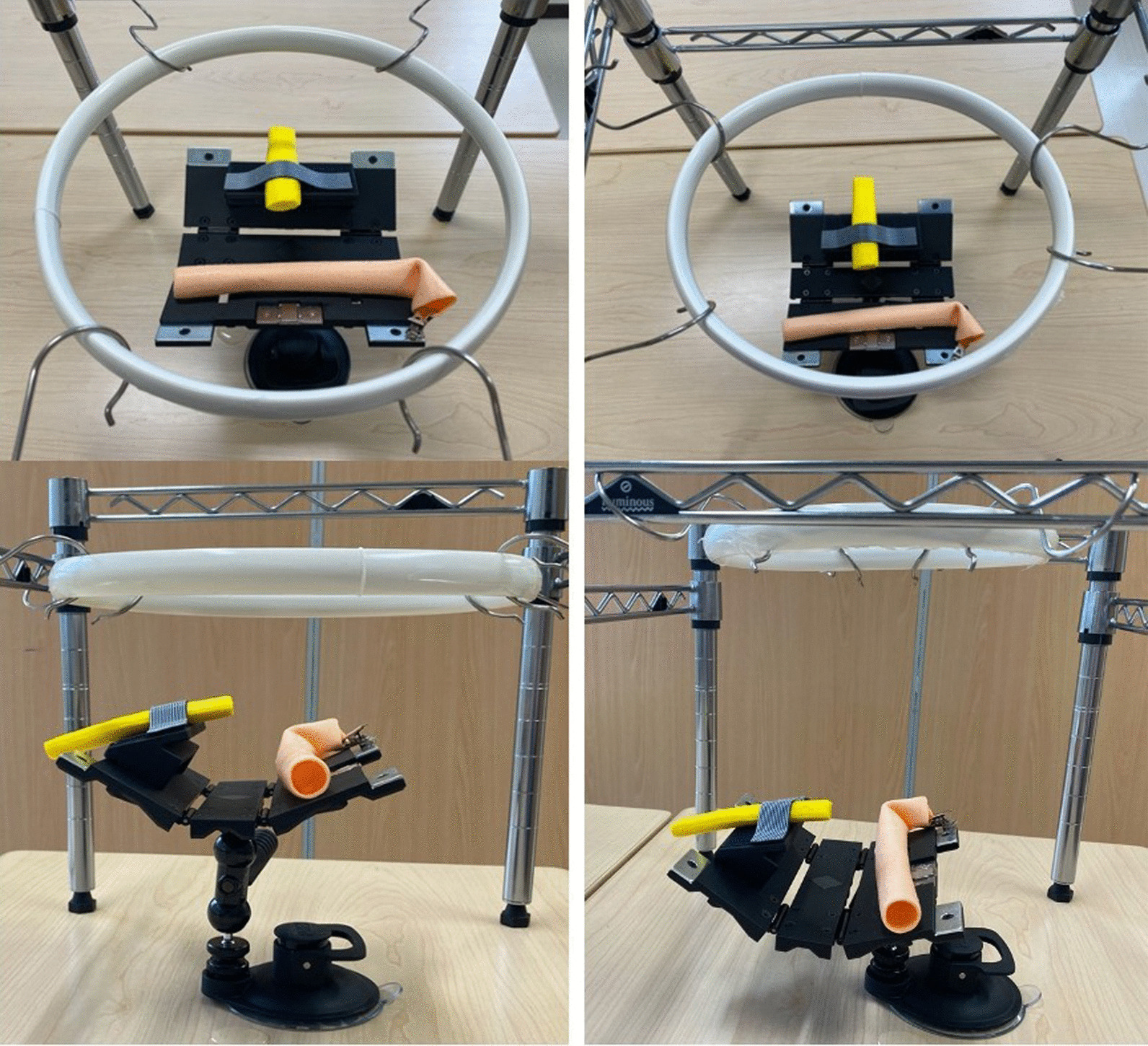
Controlling the difficulty of the pancreatojejunostomy procedure by adjusting opening size and depth of the operative field. (Left) training for beginner: large opening and shallow operative field. (Right) training for advanced surgeon: small opening and deep operative field

The working environment is an important component of a simulation teaching system. There are two important factors affecting the difficulty of the actual operation. The first factor is the size of the abdominal incision. The second is the depth from top of the abdominal wall to the remnant pancreas. Small incisions and deep surgical sites make the procedure more difficult. To simulate these two factors, we created a frame box. Elastic rings are used to reproduce the size of the abdominal incision. Different size rings are used to control the size of the opening. A large ring is 22 cm and a small ring is 18 cm in diameter. To reproduce the depth of the surgical site from the abdominal wall to the target organs, we control the height of both the ring and the model with the frame box and the fixed base (Fig. 3).

### Pilot study using this structured teaching program

Three residents (PGY3-5) and three fellows (PGY6 or above) in our department were directly recruited for this pilot study. Written consent was obtained from all participants. The procedure manual was distributed 1 week before the simulation training session. In the session, participants first received a lecture from an instructor. After that, all participants practiced PJ anastomosis training as primary surgeon under the supervision of an instructor.

As an objective assessment, the time to finish one simulation session of a PJ anastomosis was recorded. In addition, the instructor evaluated the skill of the participant using a scoring chart based on the objective structured assessment of technical skill (OSATS) examination [6, 7]. The points evaluated using a 5-point rating system included respect for tissue, instrument handling, knowledge of specific procedure and flow of operation.

Subjective assessments were carried out anonymously. Surveys about the confidence to perform PJ anastomosis in the OR were conducted before and after the session with a 5-point Likert scale (1 = strongly disagree, 2 = disagree, 3 = neutral, 4 = agree, 5 = strongly agree). After the session, participants’ opinions about the program were also evaluated with same scale.

The results of objective assessments were compared between the resident-group and the fellow-group. The confidence to perform PJ was compared between before and after the simulation training session. Median values ware used to assess the scores because of the small sample size.

## Results

### The structured program for teaching the PJ anastomosis

#### Self-learning with the procedure manual

Trainees start by learning about PJ by reading the procedure manual by themselves. The trainees can acquire both general knowledge and specific knowledge. Risk factors and early diagnosis and treatment of postoperative pancreatic fistula are especially important aspects of general knowledge. In the specific knowledge section, the standardized process was divided into twelve steps. There are clear purposes and tips discussed for each step. Trainees can acquire specific knowledge for each step with text, numbers and illustrations as preparation for simulation training.

#### Simulation training with an instructor using physical models (Additional files 1, 2 and 3)

**Fig. 4:**
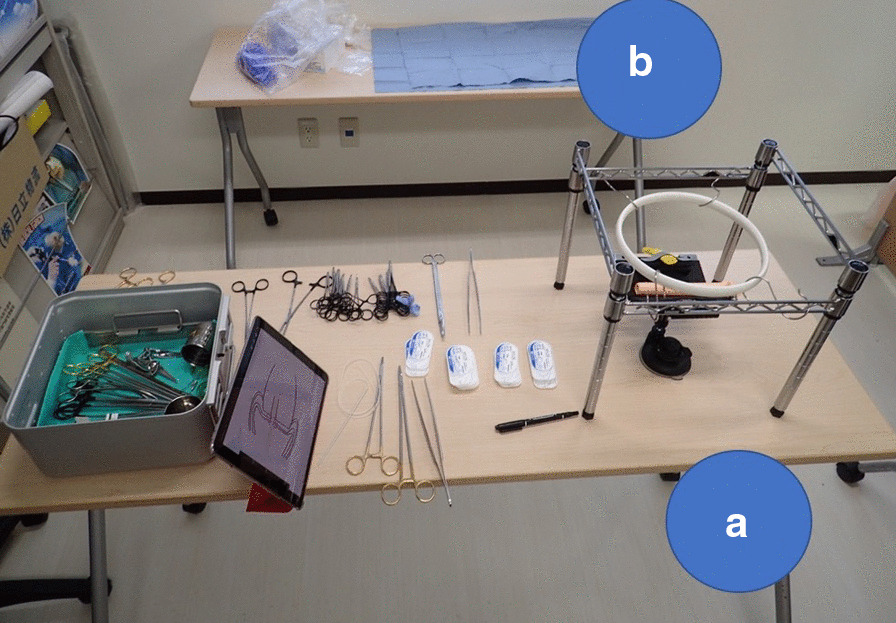
Setting of materials for simulation training of pancreatojejunostomy. **a** position for the primary surgeon. **b** position for the assistant surgeon. Trainees can assume both roles during training

We conducted simulation training outside the OR using the training materials created for this system. Training was performed with participants referring to the procedure manual on a tablet screen while performing the simulation training. A trainee and a trainer worked together and took the roles of primary surgeon and assistant surgeon alternately (Fig. 4).

**Fig. 5:**
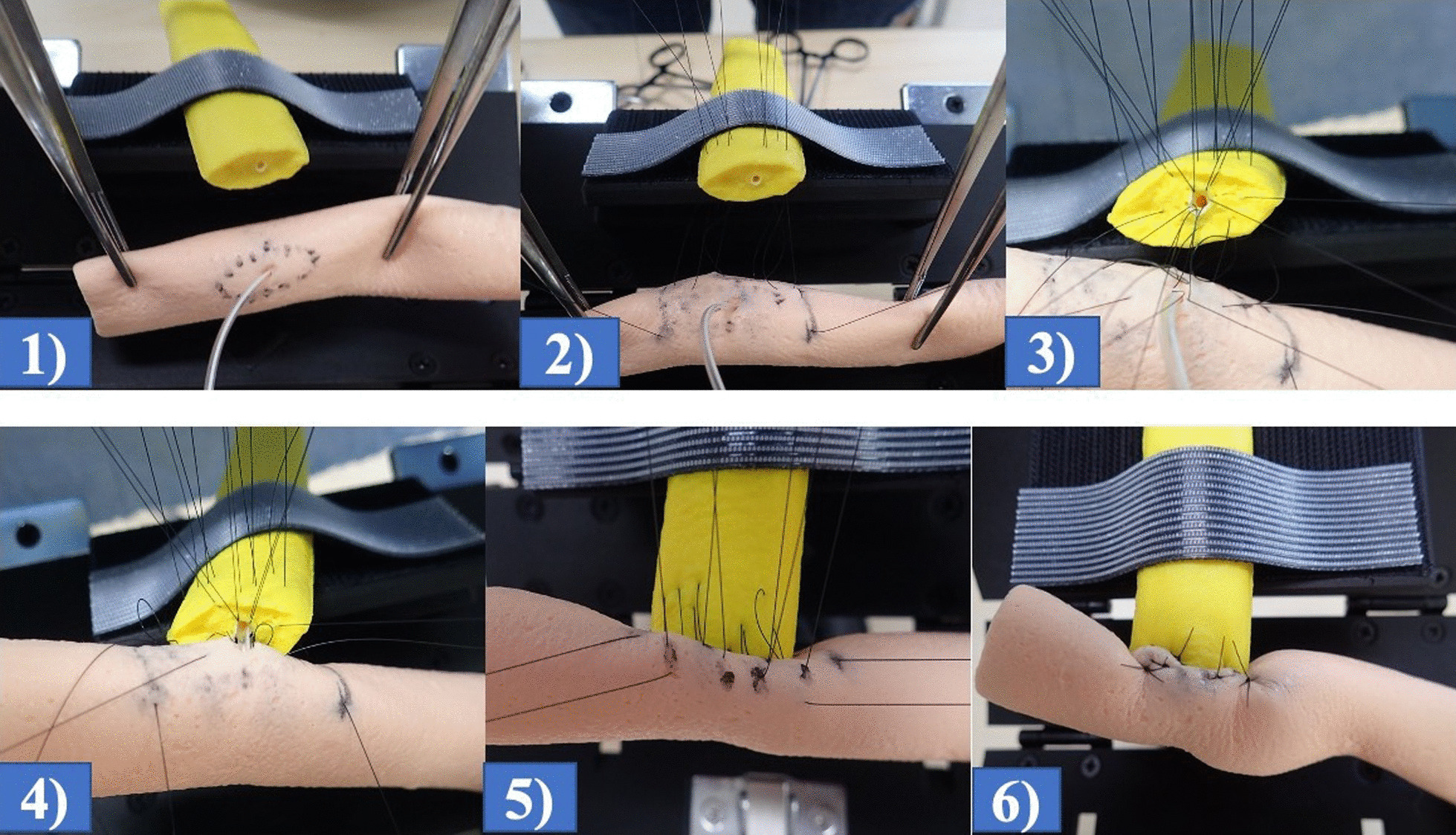
Steps of simulation training of pancreatojejunostomy. **(1)** The anastomotic site was marked, and a pancreatic drainage tube inserted through the hole in the small bowel for the anastomosis with the pancreatic duct. **(2)** Five U-shaped sutures on the posterior side of the small bowel and the pancreatic parenchyma were placed. **(3)** Duct-to-mucosa anastomosis at eighth point. First, three sutures were placed in the ventral side of the pancreatic duct to open the pancreatic duct. After that, five sutures were placed through pancreatic duct and small bowel on the dorsal side. The surgeons must be careful not to entangle the sutures. **(4)** After tying the three sutures on posterior side, the drainage tube was inserted into the pancreatic duct. **(5)** To wrap the stump of the pancreas, sutures were placed on the anterior wall of the small bowel. **(6)** After ligating the sutures, the pancreatojejunostomy was completed

**Fig. 6:**
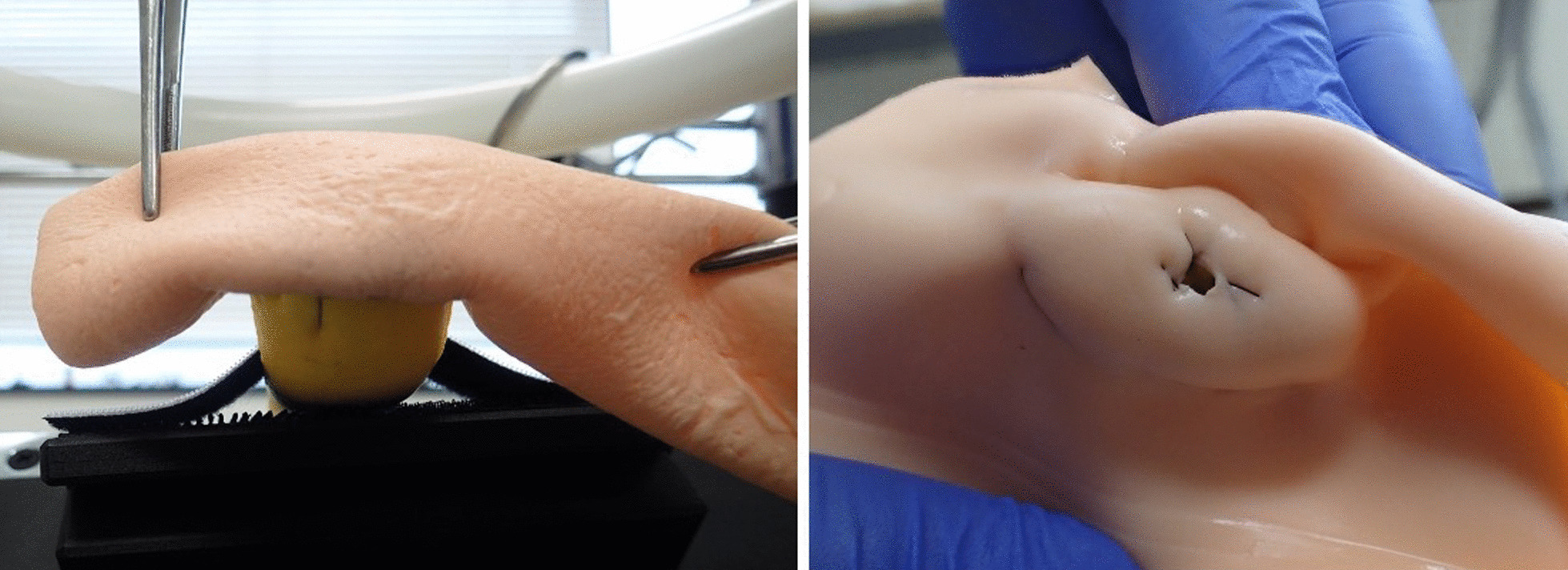
Views of the anastomotic site. (Left) View from Posterior side. (Right) View from inside of the small bowel. Please note adequate invagination of the pancreas into the small bowel and large opening of duct-to-mucosa anastomosis

Standard surgical instruments were used for the simulation training. The trainer asked the trainee if anything was unclear, and the trainer gave the trainee advice and feedback at each step of the procedure in a highly interactive manner (Fig. 5). After finishing an anastomosis, the trainee could observe the view from posterior side and from inside the small bowel, which cannot be observed in the actual operation. This observation offered an important educational benefit of using realistically designed physical models in a simulation (Fig. 6).

### Result of the pilot study (Additional files 4, 5 and 6)

**Fig. 7:**
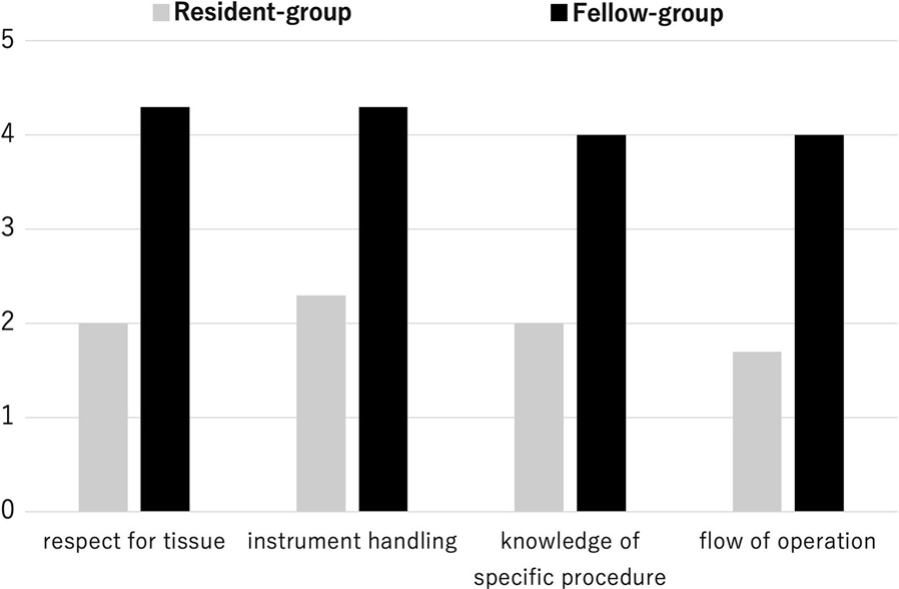
Objective assessments by the instructor. The scores of the fellow-group are higher than them of the resident-group in all four points

As objective assessments, the average times to finish a simulation training with one anastomosis were 47.6 min in the resident-group and 36.0 min in the fellow-group respectively. Evaluations by the instructor, the fellow-group was rated higher the than the resident-group in all four points (Fig. 7).

Subjective assessments were performed using questionnaires with a 5-point Likert scale. The confidence of participants to perform the PJ anastomosis in the OR increased after the simulation training (1.6–2.6). Participants in both groups considered that the teaching manual and simulation training are useful (4.2 and 4.6). They also considered that the organ models and surgical field of the simulation training are highly realistic (4.2 and 4.2) (Table 2).

**Table 2:** Pilot study results

*Demographics of the participants*	
Age (years)	34 (28–42)
Gender (male)	4 (67%)
Prior experience with pancreatoduodenectomy (as primary surgeon) (times)	3.5 (0–15)
Prior experience with PJ anastomosis (as primary surgeon) (times)	2.7 (0–13)
*Results of simulation training*	
Time to complete the PJ anastomosis	
Resident group (range)	47.6 min (42–57)
Fellow group (range)	36.0 min (33–39)
*Subjective evaluation*	
Confidence to perform PJ anastomosis method in the OR (*before* simulation training)	1.6 (1–4)
Confidence to perform PJ anastomosis method in the OR (*after* simulation training)	2.6 (1–4)
Usefulness of the manual	4.2 (2–5)
Usefulness of the simulation training	4.7 (4–5)
Reality of the organ models	4.2 (3–5)
Reality of the surgical field	4.2 (3–5)

Data for subjective evaluation are presented as median (range) using 5-point Likert scale. (1 = strongly disagree, 2 = disagree, 3 = neutral, 4 = agree, 5 = strongly agree)

*PJ* pancreatojejunostomy, *OR* operating room

## Discussion

In this study, we describe a structured program for teaching the PJ anastomosis to surgical residents and fellows. As teaching materials, we created a procedure manual, inanimate models and a frame box. From the results of this pilot study, participants greatly appreciated the structured program. The confidence of participants increased after the simulation training. The fellow group scored higher than the resident group in the subjective evaluation by the instructor.

In surgical education, training in the OR is essential. Especially, teaching difficult and complex procedure is mainly performed in the OR. However, we also need to be aware of problems associated with training in the OR. The first problem is limited teaching opportunities. Especially for difficult and complex procedures such as the PJ anastomosis, teaching opportunities where trainees perform the procedures are very limited because of the limited number of cases and considerations for patient safety. Second, the clinical situation varies greatly among cases. For the PJ anastomosis, the actual procedure may differ depending on the characteristics of the remnant pancreas. The environment of the surgical field also affects the procedure. Due to these problems, effective teaching is difficult if we only rely on the training in the OR.

Teaching outside the OR is required to overcome these problems. To perform any surgical procedure safely, both didactic knowledge and technical skills are essential. To date, structured programs for teaching both of these aspects were inadequate. We created a structured program for teaching the PJ anastomosis that combines self-learning and simulation training to complement teaching in the OR.

To create an efficient educational program, standardization is important. However, there are many kinds of techniques used to perform a PJ and there is no single technique that has emerged as the “gold standard” for PJ. Using a standardized technique and consistent practice of that single technique may lead to a decreased rate of complications [8, 9]. However, defining that standard technique remains problematic. In this study, we first created a detailed procedure manual for the PJ technique most commonly used in our institution because there were some variations in the technique even among surgeons at the same institution. Basic agreement was obtained by repeated anonymous questionnaires to obtain a consensus of the best way to teach PJ. This approach was embraced by the faculty and they were reassured that everyone’s opinion would be considered in defining the best way to teach PJ, rather than everyone following the ideas of one person. The purpose and tips for each step were verbalized, digitized and illustrated to improve the trainee’s understanding.

Standardization is also important for technical skill training. To teach surgical skills, simulation training is very useful [10–12]. There are various kinds of simulation training used outside the OR, such as virtual reality simulators, animal lab training and cadaver training. Each method has advantages and disadvantages. Dry lab simulation training has many advantages. It allows the trainee to train repeatedly in a uniform environment without ethical issues. With the recent development of three-dimensional modeling technology, it is possible to create models to use for training complex procedures [13–15]. We used this technology to develop the training models for PJ. These models reproduce human anatomy with great accuracy to facilitate PJ training. These teaching models made it possible to teach suturing and ligation on anatomically accurate models, which can be used multiple times.

The difficulty of PJ is influenced by access through the abdominal incision and depth of the surgical field. Especially, the depth of the operative field is a risk factor for the development of postoperative pancreatic fistula [16]. The influence of both factors on training for PJ can be adjusted with this teaching system.

Based on the results of the pilot study, all participants responded that this program was useful. Furthermore, the confidence to perform PJ anastomosis increased after simulation training. We consider that our program will be a good start for the training in the OR. The objective evaluation showed a difference in the score according to the experience of the surgical training. Objective assessment by the instructor might be used as an indicator of readiness for training in the OR. It is desirable for trainees who are prepared up to a certain level to receive further training in the OR.

There are two major issues which remain to be solved. The first issue is the cost of the program. The dry lab training model set including the pancreas and the small intestine costs about US$100 to produce. It is possible to use one set for four PJ anastomoses. The needles cost about US$70 for a single anastomosis. Therefore, it costs about US$380 (set of models plus needles) to perform four anastomoses. We want to reduce it to less than US$100 (US$25 per anastomosis). Further cost reductions may be possible with mass production and devising new materials. The second issue to be solved is to further examine the teaching effect of this program. There are many studies about usefulness of the simulation training in minimally invasive surgery, however evaluation in open surgery is still inadequate [17–19]. In this pilot study, an evaluation chart based on OSATS were used and results were obtained from the perspective of evaluation technical skills. This was a pilot study with a few participants which precluded a detailed statistical analysis of the results. It is necessary to carry out further large-scale studies in the future.

## Conclusion

We have developed a simulation program for teaching PJ. This training outside the OR is expected to complement surgical training in the OR.

## Supplementary Information

**Additional file 1.** Include movies of simulation training for pancreatojejunostomy with our modified Blumgart technique.

**Additional file 2.** Include movies of simulation training for pancreatojejunostomy with our modified Blumgart technique.

**Additional file 3.** Include movies of simulation training for pancreatojejunostomy with our modified Blumgart technique.

**Additional file 4.** Questionnaire for subjective assessment (before simulation training).

**Additional file 5.** Questionnaire for subjective assessment (after simulation training).

**Additional file 6.** Objective assessment completed by the instructor.

## Data Availability

All data analyzed during this study available from the corresponding author on reasonable request.

## Abbreviations

PJ: Pancreatojejunostomy
OR: Operating room
