# Supplementary material for: A structured program for teaching pancreatojejunostomy to surgical residents and fellows outside the operating room: a pilot study
Source: BMC Surg. 2021 Feb 25;21:102. doi: 10.1186/s12893-021-01101-w (PMC7908720; doi:10.1186/s12893-021-01101-w)
Supplement: Supplementary file 4 — Additional file 4. Questionnaire for subjective assessment (before simulation training). [file 12893_2021_1101_MOESM4_ESM.docx]

Participant Questionnaire (**Before simulation training**)

**Participant No. ( )**

**Q1) Age ( ) y.o.**

**Q2) Gender ( Male or Female )**

**Q3) Post graduate year (PGY) ( )**

**Q4) Have you performed a pancreatoduodenectomy as primary surgeon? If yes, how many have you done?**

**( Yes ( ) cases, or No )**

**Q5) Have you performed pancreatojejunostomy as primary surgeon? If yes, how many have you done?**

**( Yes ( ) cases, or No )**

**Q6) I am confident to perform a pancreatojejunostomy by myself.**

**( 1 2 3 4 5 )**

(1= not confident at all, 2=slightly confident, 3=somewhat confident, 4=fairly confident, 5=completely confident)
