# Supplementary material for: A structured program for teaching pancreatojejunostomy to surgical residents and fellows outside the operating room: a pilot study
Source: BMC Surg. 2021 Feb 25;21:102. doi: 10.1186/s12893-021-01101-w (PMC7908720; doi:10.1186/s12893-021-01101-w)
Supplement: Supplementary file 5 — Additional file 5. Questionnaire for subjective assessment (after simulation training). [file 12893_2021_1101_MOESM5_ESM.docx]

Participant Questionnaire (**After simulation training**)

**Participant No. ( )**

**Q1) The procedure manual is useful for learning pancreatojejunostomy using the modified Blumgart method.**

**( 1 2 3 4 5 )**

(1=strongly disagree, 2=disagree, 3=neutral, 4=agree, 5=strongly agree)

**Q2) The simulation training is useful for learning pancreatojejunostomy using the modified Blumgart method.**

**( 1 2 3 4 5 )**

(1=strongly disagree, 2=disagree, 3=neutral, 4=agree, 5=strongly agree)

**Q3) The organ models are realistic.**

**( 1 2 3 4 5 )**

(1=strongly disagree, 2=disagree, 3=neutral, 4=agree, 5=strongly agree)

**Q4) The operative field is realistic.**

**( 1 2 3 4 5 )**

(1=strongly disagree, 2=disagree, 3=neutral, 4=agree, 5=strongly agree)

**Q5) I am confident to perform a pancreatojejunostomy by myself.**

**( 1 2 3 4 5 )**

(1= not confident at all, 2=slightly confident, 3=somewhat confident, 4=fairly confident, 5=completely confident)
