# Supplementary material for: A structured program for teaching pancreatojejunostomy to surgical residents and fellows outside the operating room: a pilot study
Source: BMC Surg. 2021 Feb 25;21:102. doi: 10.1186/s12893-021-01101-w (PMC7908720; doi:10.1186/s12893-021-01101-w)
Supplement: Supplementary file 6 — Additional file 6. Objective assessment completed by the instructor. [file 12893_2021_1101_MOESM6_ESM.docx]

Objective Evaluation Sheet (for the instructor)

**Participant No. ( )**

**Time to complete the PJ anastomosis ( ) min.**

Rate the participant’s performance on the following points:

**Point 1) Respect for tissue**

**( 1 2 3 4 5 )**

1= Frequently used unnecessary force on tissue or caused damage by inappropriate use of instruments.

3= Careful handling of tissue but occasionally caused inadvertent damage.

5= Consistently handled tissues appropriately with minimal damage.

**Point 2) Instrument handling**

**( 1 2 3 4 5 )**

1= Frequently makes tentative or awkward moves with instruments.

3= Competent use of instruments although occasionally appeared stiff or awkward.

5= Fluid moves with instruments and no awkwardness.

**Point 3) Knowledge of specific procedure**

**( 1 2 3 4 5 )**

1= Deficient knowledge. Needed specific instruction at most operative steps.

3= Knew all important aspects of the operation.

5= Demonstrated familiarity with all aspects of the operation.

**Point 4) Flow of operation**

**( 1 2 3 4 5 )**

1= Frequently stopped operating or needed to discuss next step.

3= Demonstrated ability for forward planning with steady progression of operative procedure.

5= Obviously planned course of operation with effortless flow from one move to the next.
